# Histological response of peritoneal carcinomatosis after hyperthermic intraperitoneal chemoperfusion (HIPEC) in experimental investigations

**DOI:** 10.1186/1471-2407-6-162

**Published:** 2006-06-22

**Authors:** Joerg OW Pelz, J Doerfer, A Dimmler, W Hohenberger, T Meyer

**Affiliations:** 1 Department of Surgery, University of Erlangen-Nuremberg, Germany; 2 Institute of Pathology, University of Erlangen-Nuremberg, Germany

## Abstract

**Background:**

In selected patients with peritoneal carcinomatosis from colorectal cancer prognosis can be improved by hyperthermic intraperitoneal chemotherapy (HIPEC) after cytoreductive surgery. The aim of this study was to evaluate the tumor response of peritoneal carcinomatosis in tumor-bearing rats treated with HIPEC.

**Methods:**

CC531 colon carcinoma (2,5 × 10^6 ^cells), implanted intraperitoneally in Wag/Rija rats, was treated by hyperthermic intraperitoneal chemotherapy. After 10 days of tumor growth the animals were randomized into five groups of six animals each: group I: control (n = 6), group II: sham operated animals (n = 6), group III: hyperthermic intraperitoneal perfusion (HIP) without cytostatic drugs, group IV: HIPEC with mitomycin C in a concentration of 15 mg/m^2 ^(n = 6), group V: mitomycin C i.p. alone in a concentration of 10 mg/m^2 ^(n = 6). After 10 days the extent of tumor spread and histological outcome were analysed by autopsy.

**Results:**

All control animals developed extensive intraperitoneal tumor growth. Histological tumor load was significantly reduced in group III and group V and was lowest in group IV. In group II tumor load was significantly higher than in group I. Implanted metastases were significantly decreased in group IV compared with group I and group II.

**Conclusion:**

These findings indicate that HIPEC is an effective treatment for peritoneal carcinomatosis in this animal model. HIPEC reduced macroscopic and microscopic intraperitoneal tumor spread.

## Background

Gastrointestinal malignancies frequently recur with metastatic disease limited to the abdominal cavity. The peritoneal failure rate among patients who present with recurrence after colon cancer resection is approximately 25–35% [1].

Peritoneal dissemination of colon cancer cells is a common cause of morbidity and mortality in recurrent disease that may result in intestinal obstruction, ascites and intestinal fistula. The median survival time after manifestation of peritoneal carcinomatosis is about 6–9 months [2].

Peritoneal seeding from colorectal cancer is relatively resistant to systemic chemotherapy. A treatment strategy for these patients would be hyperthermic intraperitoneal chemotherapy (HIPEC). The first clinical hyperthermic chemotherapy in the treatment of peritoneal carcinomatosis (PC) was performed by Spratt et al. in 1980 [3]. This therapeutic design is regarded as one of the best options for the therapy of peritoneal metastasis from gastrointestinal carcinoma. The feasibility of intraperitoneal therapy has been demonstrated by several groups which used this technique in combination with cytoreductive surgery to treat peritoneal carcinomatosis [4,5].

Macroscopic complete resection of PC followed by HIPEC is potentially capable of curing selected patients presenting with disease confined to the peritoneum. The goal of cytoreductive surgery is to achieve a nearly total resection of all tumor tissue. In selected patients, HIPEC may lead to a five-year overall survival of 27% in PC [6].

Although the technique of hyperthermic intraperitoneal chemoperfusion in humans has been employed in cancer therapy. In clinical trials human populations are very heterogeneous and animal models potential differences are less. In this study, HIPEC was evaluated in an experimental tumor bearing rat model.

## Methods

### Animals

To investigate HIPEC a rat model was used first described by Martin et al. and modified in our research group [7].

Maintenance and care of all experimental animals were carried out according to the guidelines of the local responsible Animal Protection Commission and carried out in compliance with national guidelines (National Institute of Health for Use of Laboratory Animals; Nr. 621-2531.31-5/03). 30 inbred male pathogen-free WAG (Wistar Albino Glaxo) rats (Charles River, Sulzbach, Germany) of reproductive age weighing 200 to 240 g, (Sulzfeld, Germany), were used in this study. They were fed a standard laboratory diet and tap water *ad libidum*. The animals were kept in individual cages during the experiment with a 12 hours light and dark cycle and room temperature of 25°C, with a relative humidity of 55 per cent.

### Tumor model

The tumor cell line used (German Cancer Research Centre, Heidelberg, German)y was an adenocarcinoma of the rat colon. These immunocompetent tumor cell line (CC531) was derived from a G2 differentiated colon carcinoma induced by 1,2-dimethylhydrazine [8].

To control for possible mutations of cell lines, only cultures that had undergone less than 10 passages were used in the experiments. Intraperitoneal tumor application was performed with a tumor suspension produced in vitro. The tumor cell line was cultivated at 37°C and 5% CO_2 _in an incubator in 20 ml complete medium (RPMI 1640 [Gibco, Life Technologies, Eggenstein, Germany], 10% fetal bovine serum [Seromed, Biochrom, Berlin, Germany] and 1% Penicillin/Streptomycin [Seromed]). After three days, cells were detached with 3 ml trypsin (0.25%). Vitality was evaluated in a Bürker hematocytometer after the addition of trypan blue. Viability always exceeded 95 per cent. After vital counting, the suspension had a density of 2,5 × 10^6 ^vital cells/200 μl suspension before being injected into the animals.

In all rats, tumor cell implantation was achieved via a 6 cm laparotomy. The rats were anaesthetized by Isoflurane inhalation (Baxter, Unterschleißheim, Germany). The tumor cell suspension was injected under the capsule of the peritoneal surface in the right upper side of the abdomen as described before [7].

The animals were randomised into five groups of six animals each:

group I: control

group II: sham operated animals

group III: hyperthermic intraperitoneal perfusion (HIP) without cytostatic drugs,

group IV: HIPEC with mitomycin C in a concentration of 15 mg/m^2^

group V: mitomycin C i.p. alone in a concentration of 10 mg/m^2^

10 days after tumor inoculation, the animals from group II-V underwent a further laparotomy as described. In group II and III perfusion was performed. In group II the tumor remainded untreated. In group V mitomycin C was aplivated intraperitonally in 5 ml saline. Saline was not removed. Abdomen was open for 90 minutes in groups III and V.

Compared to the clinical situation, the MMC ratio in our experimental trial was 1,5:1 (HIPEC versus i.p. therapy). This was performed as discribed before [7].

### Perfusion system

The HIPEC system is a closed circuit, allowing perfusate circulation with a variable dynamic flow of 40–50 ml/minute. The warmed perfusate (500 ml) was driven by a roller pump with two synchronously running pump-heads on a single axis for the inflow and the outflow lines (Masterflex^®^). An inflow catheter was inserted into the upper abdomen between the hepatic and diaphragmatic surface, and an outflow catheter was placed within the pouch of Douglas.

The intraperitoneal temperature was maintained between 40,5 and 41°C. Baseline temperature was recorded for 5 minutes before treatment. Temperature was continuously measured during application. In group I (control), group II (sham operated group) and group V (MMC only) temperature measurement was not performed.

Perfusion was performed over 90 minutes after the perfusion fluid had reached the required temperature. The body surface of the animals was calculated according the formula (A(m^2^) = m_k_^0,425 ^× 1_K_^0,725^/139.315 (m^2 ^= body surface; m_k _= body lenght 1_K _= body weight). In group IV, mitomycin C was added to the perfusate in three divided doses at 30 min intervals in a drug concentration of 15 mg/m^2^. The first dose contained 50% and the following administrations 25% of the total dose.

After the perfusion the perfusate was removed and the abdomen was irrigated with sodium chloride for 10 minutes. Afterthere, the abdomen was closed in two layers.

### Evaluation

Postoperatively, all animals were kept in individual cages. The animals were kept under standard conditions and were sacrificed by an overdose of anesthetics and cervical dislocation on the 10^th ^postoperative day.

All animals were autopsied and peritoneal carcinomatosis was evaluated qualitatively and quantitatively. The macroscopic tumor nodules were counted. The tumor weight was determined using a digital balance.

Conventional histology using H&E staining classified specimens for occurrence of metastases.

### Clinical tumor response

Intraperitoneal tumor growth was scored with a semiquantitative cancer index [9]. The scoring ranged from 0 to 5 and was performed by 2 independent observers. A score of 0 meant that there was no tumor growth; a score of 1 indicated an estimated tumor diameter less than 0.5 cm; a score of 2, a tumor diameter between 0.5 and 1 cm; a score of 3, a tumor diameter between 1 and 2 cm; a score of 4, a tumor diameter between 2 and 3 cm; and a score of 5, a tumor diameter of more than 3 cm.

### Histologic determination of apoptotic indices of cancer

To determine the extent of apoptosis in the tumor, samples were fixed in 10% buffered formaline (pH 6,9–7.1). From each paraffin-embedded sample, 5 μm thick sections were prepared and stained with hematoxylin and eosin (H&E) for light microscopic examination. The 3'-end labelling of apoptotic cell DNA was performed with an ApopTag in situ apoptosis kit according to the recommended procedures of the Kit (Roche Diagnostics, Mannheim, Germany (in situ cell death detection kit)).

The mean number of apoptotic cells and bodies in the entire cancer cell population was determined by counting their numbers in 5 high-power fields of non-necrotic areas. The index represented the number of visible apoptotic cancer cells in these fields.

### Statistical analysis

Data were analyzed using SPSS/PC+ statistical software. The mean scores were calculated. All data are presented as the mean value and standard deviation errors in the single groups. For comparison of tumor volume and microvessel density between the diffent groups a non-parametric test was used (Kruskal-Wallis). Differences were considered significant at a calculated *p *value of less than 0.05.

## Results

The location and diameter of peritoneal metastasis did not differ significantly among the four treatment groups (II-V) at the time of treatment. Before the intervention diameter of the treated tumors was 5 mm. There were only one tumor in all cases.

Neither sudden deaths occurred postoperatively nor did any animal had to be sacrified because of adverse effects. All animals could be evaluated 10 days following surgery.

### Tumor load

In group I, all animals developed extensive intraperitoneal tumor growth. In group IV, no macroscopic tumor were found in 2 rats, in three rats only locally limited metastases were found. These tumors were significantly smaller than in all other groups (p < 0,02). No diffuse peritoneal spread was found in group IV.

The mean values of peritoneal cancer index in group I was 3,5. In group II the incidence of metastases spread was significantly higher than in group I (p < 0.05). Diffuse peritoneal carcinomatosis was found in all animals of group I and group II. The cancer indices were significantly lower in group III and in group V compared with group I. The lowest tumor load was observed in group IV. No significant differences in total tumor load was found between group III and group V.

**Figure 1:**
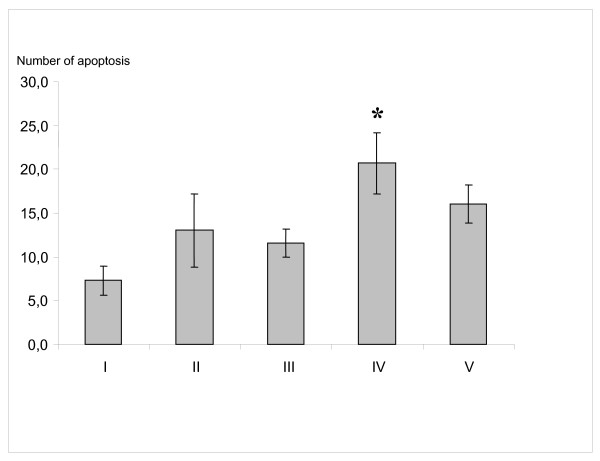
The mean number of apoptotic cells in 5 high-power fields of non-necrotic areas. (group I: control; group II: sham operated animals; group III: hyperthermia alone; group IV: HIPEC; group V: MMC i.p. alone) * p < 0.036 group IV versus group I; (Kruskal-Wallis test)

The tumor scores from group I-V are given in Fig 1.

### Tumor weight

Median total tumor weight (local metastases and tumor spread) in group I were 8,1 g ± 3,4 g. The median weight of total tumor in group II was significantly higher (16,4 g ± 5,6 g). Total tumor weight in group III and group V was significantly lower (5,9 g ± 2,8 g and 5,7 g ± 2,4 g, respectively). Lowest total tumor weight was found in group IV (1,8 g ± 0,9 g mg) (P < 0.05). There was no difference in tumor weight in group III and group V.

Tumor median nodules 10 days after intervention were 35 ± 12 in group I and 68 ± 17 in group II (p < 0.05). The Significantly lowest number of tumor nodules were found in group IV with 4 ± 7. In group III and group V tumor nodules were 21 ± 9 and 16 ± 10, respectively.

The median amount of ascites in the control group (3/5 animals) was 1,34 ml (0–3,2 ml), and 3,21 ml (1,2–4,9 ml) in group II (5/5 animals) was. Ascites was not detected in any animal in group III, IV and V.

### Clinical response

In groups I, II and III in all six animals tumor growth was seen macroscolicaly. In group V 1/6 animals showed a complete response of disease. In group IV 4/6 animals had a clinically total tumor remission.

### Histologic findings

In conventional histology (H&E), the tumors in groups I and II showed no morphological differences. Tumors were found to be adenocarcinoma with a moderate differentation, mainly tubular structures, vacuolar lumen formation and fibrous septa. Spontaneous necrosis rate ranged between aproximately 30 and 40%. Tumor morphology did not change with the tumor size.

In group IV the tumors showed signs of irreversible cell damage after treatment. Tumor cells displayed clear shrinkage and partial loss of cell contact. Thromboses of the larger adjacent vessels were found on the tumor-muscle border. Infiltration with macrophages was present in groups III and IV, but was more pronounced in group IV. In group III there were less signs of irreversible cell damage after treatment than in group IV (Table 1).

In group I, II and III and V in all animals vital tumor was found. In group IV in 4/6 animals vital tumor was found. The zones of viable tumor cells were located in the center of the tumor. The zones of apoptosis in group IV was about 3 mm in mean from the tumor margin. Two animals out of six showed no vital tumor. In groups III and V no clear zones were seen.

**Figure 2:**
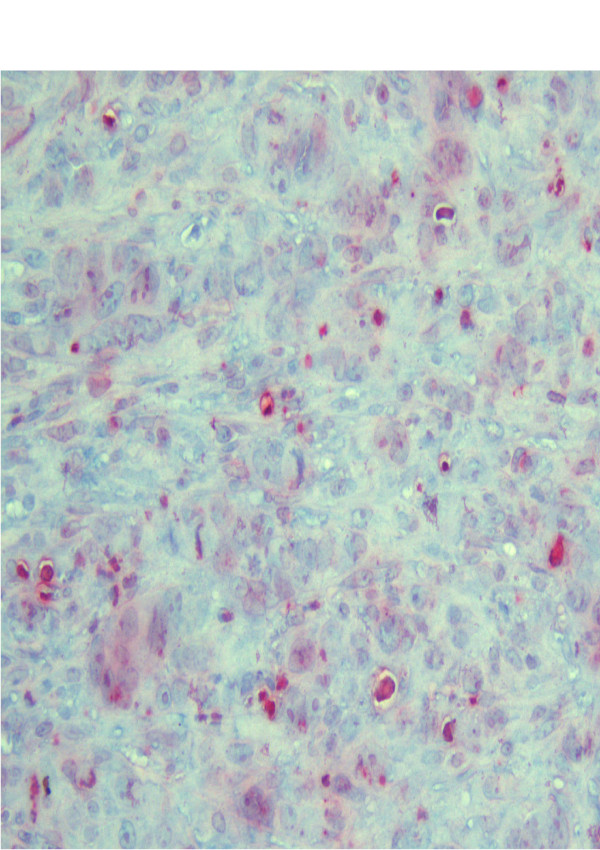
Immunohistochemical staining for group IV (HIPEC): Apoptosis are represented by red clusters. The index represented the number of visible apoptotic cancer cells in five fields.

**Figure 3:**
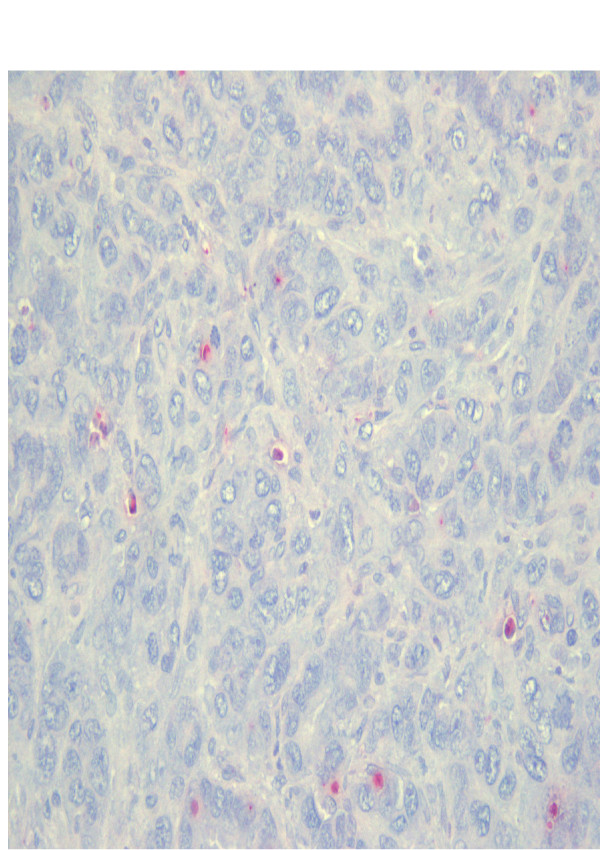
Immunohistochemical staining group I (control): Apoptosis are represented by red clusters. The index represented the number of visible apoptotic cancer cells in five fields

Table 1 shows the kinetics of tumor apoptosis induced by each treatment. The semiquantitative assessment of immunhistological stainings revealed a significantly higher apoptosis level in group IV (Fig. 2) compared to group I (Fig. 3) and II 10 days after intervention (p = 0,01). The maximum of induced apoptosis was highest in group IV (p < 0.036). The apoptotic cells located widespread in the tumor margin.

Different expression levels in group III and group V were not detectable after 10 days (p > 0,05)

## Discussion

Peritoneal carcinomatosis represents a large problem in the treatment of colorectal carcinomas. A new therapeutic option is HIPEC. The advantage of HIPEC is that thecytostatic drug, unlike systemic chemotherapy, can be delivered directly into the abdominal cavity. The hyperthermic aspect of the therapy results in an additive effect for the destruction of tumor cells.

Recent clinical findings suggest that HIPEC is a very promising therapeutic option. Many authors were able to demonstrate improved chances for survival in selected patient groups [10-14]. Clinical trials are, however, based on non-homogenous and non-standardized groups. Additionally, the only possibilities for evaluating actual tumor response are indirect, through imaging procedures and survival rates.

Because of the promising clinical results, it is worthwhile to study HIPEC under experimental conditions. The primary parameters would be the histological and morphological changes in peritoneal carcinomatosis after HIPEC.

A previously published study of locally limited peritoneal carcinomatosis in a rat model, in which HIPEC could be performed with long term survival [7], provided the basis for these experiments. This tumor model is very similar in its biological traits to that of human colorectal carcinomas.

A phenomenon that has been described is the rapid increase of tumor growth following surgical manipulation.

This was also observed in our sham-operated group of animals in which tumors grew more rapidly than in the control group. This implies that resection is associated with an accelerated growth in residual tumors. It is assumed that the surgical manipulation induces liberation of growth factors that in addition to their effect on healing also have a stimulating effect on tumor proliferation.

In order to better examine the individual aspects of the effects of HIPEC, two groups were added in addition to group IV; these groups investigated the two noxa (cytostatics and hyperthermia) individually (group III: hyperthermic perfusion without MMC; group V: MMC as mono-therapy). Two control groups were used for comparison with the treatment groups, so that the natural progression of peritoneal carcinomatosis could be pursued. To come as close as possible to the clinical situation, the same parameters were used for the experimental perfusions as are used in clinical treatment. The only difference was the lower dose of MMC, as other research groups have already shown that the experimental tumor can be completely eradicated by using an equivalent dose. The ratio of clinical MMC dose HIPEC/mono-therapy was, however, maintained [7].

The MMC dose was not set too high, as can be seen from our results. Thus, a therapeutic effect could be recognized in group 5, although all animals did experience clinical tumor progression. Histological results showed that in addition to areas of tumor necrosis and clearly apoptotic zones, there were also many areas with viable tumor cells. It was not possible to destroy the tumor in any animal completely by application of MMC alone.

Similar results were observed in group III. This group also showed a significant slowing of tumor growth and development of peritoneal. However, tumor regression could be observed in all animals in this group. Histological results were similar to those from group V and showed partial damage to the tumor tissue. Complete destruction of the tumor and thus a definitive therapy could not be achieved.

The results of groups III and V demonstrate clearly that a combination of the two components is necessary. The results from group IV show that the benefits of combination therapy are not limited to a further significant reduction in growth rate as compared to groups III and V. Complete eradication of the tumor with no histological indication of viable tumor cells was possible in two of six animals. The size of the treated tumor decreased significantly in these two. Although there was also a clinical reduction in tumor size in the other two animals, viable tumor cells could still be identified after HIPEC in these two of six animals. This supports the findings of other working groups, who showed that HIPEC is best suited for tumors smaller than 5 mm because of the limited depth of penetration [15] This size (5 mm) was the chosen tumor size in our experiments. The results emphasize the importance of performing HIPEC in a clinical setting only after radical cytoreduction.

The present study also demonstrates the limits of this new therapy. Complete eradication of tumors after experimental HIPEC was not possible in all animals. Despite definite slowing of tumor growth and inhibition of diffuse peritoneal seeding over a 10 days' period, further improvements for the optimization of HIPEC, such as new cytostatic drugs, must be sought. Moreover, the results show that clinical reduction of tumor mass is not necessarily associated with a relapse-free course of disease in this animal model.

## Conclusion

In this study the effect of HIPEC on peritoneal carcinomatosis of colorectal tumors could be investigated in a longterm standardized animal model. Results showed that tumor growth was significantly slowed and, in some cases, completely arrested. However, these experiments also showed that therapy which appears to have been clinically successful residual areas of viable tumor cells, maybe left which lead to recurrence of the disease.

## Competing interests

The author(s) declare that they have no competing interests.

## Authors' contributions

JP participated in the design carried out the treatment and drafted the manuscript.

JD carried out the treatment.

AD carried out the histological studies

WH participiated in the design of the study

TM participated in the design and coordination of the study.

All authors read and approved the final manuscript.

## Pre-publication history

The pre-publication history for this paper can be accessed here:



## Figures and Tables

**Table 1:** Tumor response in group I (control; n = 6), II (HIPEC; n = 6), and III (MMC only; n = 6) 10 days after intervention. * p < 0.05 group IV versus group I; § p < 0.05 group V versus group I (Kruskal-Wallis test)

	Group I	Group II	Group III	Group IV	Group V
Tumor weight (g)	8,1 ± 3,4	16,4 ± 5,6	5,9 ± 2,8	1,8 ± 0,9 *	5,7 ± 2,4^§^
Tumour nodules	35 ± 12	68 ± 17	21 ± 9	4 ± 7 *	16 ± 10^§^
Cancer index	3,5	4,2	2,2	1,4 *	2,6
Clinical CR	0/6	0/6	0/6	4/6	1/6
Histological CR	0/6	0/6	0/6	2/6	0/6
